# Shotgun proteomics profiling of chia seeds (*Salvia hispanica* L.) reveals genotypic differential responses to viability loss

**DOI:** 10.3389/fpls.2024.1441234

**Published:** 2024-08-15

**Authors:** María Emilia Rodríguez, Laura Poza-Viejo, Isaac Maestro-Gaitán, Aline Schneider-Teixeira, Lorena Deladino, Vanesa Ixtaina, Maria Reguera

**Affiliations:** ^1^ Centro de Investigación y Desarrollo en Criotecnología de Alimentos (CIDCA) [CONICET La Plata, Facultad de Ciencias Exactas-Universidad Nacional de La Plata (UNLP), Comisión de Investigaciones Científicas de la Provincia de Buenos Aires (CICBA)], La Plata, Buenos Aires, Argentina; ^2^ Facultad de Ciencias Agrarias y Forestales, Universidad Nacional de La Plata (FCAyF-UNLP), La Plata, Buenos Aires, Argentina; ^3^ Department of Biology, Universidad Autónoma de Madrid, Madrid, Spain; ^4^ YPF-TECNOLOGÍA (Y-TEC), Berisso, Argentina

**Keywords:** chia, nutlet, seed viability, seed longevity, artificial aging, emergent crops, proteomics

## Abstract

**Introduction:**

Exposure to elevated temperatures and relative humidity expedites the seed aging process, finally leading to seed viability loss. In this context, certain proteins play a pivotal role in safeguarding the longevity of seeds. However, the seedproteomic response to loss viability in Salvia hispanica L., commonly known as chia, remains incompletely understood.

**Methods:**

This work explores the application of proteomics as a potent tool for uncovering molecular responses to viability loss caused by artificial aging in two chia genotypes, WN and MN.

**Results:**

By using a quantitative label-free proteomics analysis (LC-MS/MS), 1787 proteins wereidentified in chia seeds at a 95% confidence level, including storage proteins, heat shock proteins (HSPs), late embryogenesis abundant proteins (LEA),oleosins, reactive oxygen species (ROS)-related enzymes, and ribosomal proteins. A relatively low percentage of exclusive proteins were identified in viable and non-viable seeds. However, proteins exhibiting differential abundancebetween samples indicated variations in the genotype and physiological status. Specifically, the WN genotype showed 130 proteins with differential abundancecomparing viable and non-viable seeds, while MN displayed changes in the abundance of 174 proteins. While both showed a significant decrease in keyproteins responsible for maintaining seed functionality, longevity, and vigor withhigh-temperature and humidity conditions, such as LEA proteins or HSPs, ROS, and oleosins, distinct responses between genotypes were noted, particularly in ribosomal proteins that were accumulated in MN and diminished in WN seeds.

**Discussion:**

Overall, the results emphasize the importance of evaluating changes in proteins of viable and non-viable seeds as they offer valuable insights into the underlying biological mechanisms responsible for the maintenance of chia seed integrity throughout high-temperature and humidity exposure.

## Graphical Abstract

Shotgun Proteomics reveals potential biomarkers for chia seed viability. The results of shotgun proteomics analysis identified potential biomarkers for chia seed viability after exposure to artificial aging (40°C temperature and 100% Relative Humidity (RH) for 56 days). Seed viability is represented by images of 2,3,5-triphenyl-2 H-tetrazolium chloride (TTZ) stained chia seeds showing red staining (viable seeds) or non-stained seed (non-viable seed). Key proteins, including those involved in storage, stress response [such as Heat Shock Proteins (HSPs) and late embryogenesis abundant proteins (LEA)], reactive oxygen species (ROS) metabolism, membrane structural integrity, energy starvation, and ribosomal proteins exhibit differential abundance in viable versus non-viable seeds. Notably, while both genotypes show significant reductions in crucial proteins under stress conditions, specific variations in ribosomal protein responses were evident.

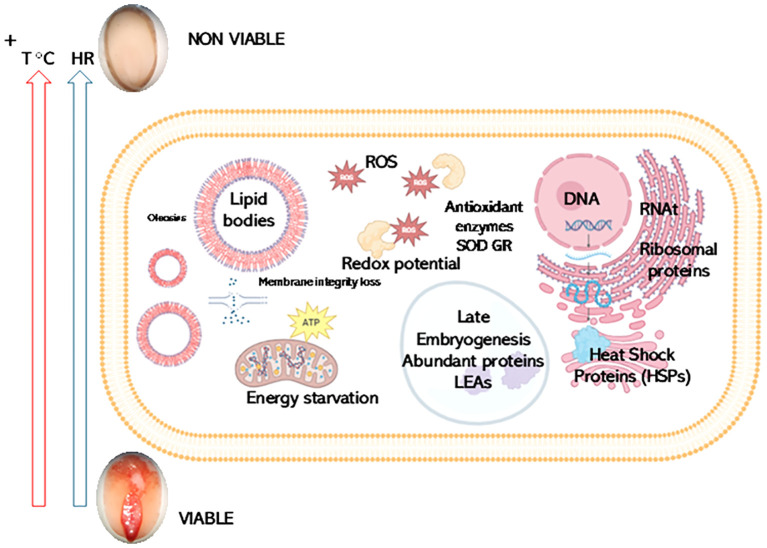

## 1 Introduction

Minor crops, often grown in specific local and regional environments, contribute to plant-based food diversity enrichment. These crops involve a genotype of fruits, vegetables, and grains that, though not widely grown on a global scale, are valuable for local nutrition, biodiversity, and cultural significance (Sytar, 2023). Expanding their use enhances agricultural biodiversity and provides genetic and species diversity (Ebert and Engels, 2020), protecting against the susceptibility of crops to climate change, pests, and diseases (Lin, 2011). Additionally, it helps ensure high-quality food availability and addresses food and nutritional security concerns (Singh et al., 2022).

*Salvia hispanica* L., commonly known as chia, has gained popularity as a nutritious food source considered a promising crop for achieving food security (Prathyusha et al., 2019). The main composition of chia seeds consists of 30–33% lipids, 15–25% protein, 18–30% dietary fiber, 41% carbohydrates, and 4–5% ash (Chaudhary et al., 2021). The seeds have a higher protein content than grains cereal crops such as oats, wheat, and rice, with globulins being the main fraction (Hrnčič et al., 2020). Also, about 80% of the lipid content of chia seeds are polyunsaturated fatty acids (PUFA), such as α-linolenic acid (C18:3, ω-3) (64%) and linoleic acid (C18:2, ω-6) (20%), which are essential in the human diet (Agarwal et al., 2023). *S. hispanica* is a native plant from Mexico and Guatemala (Wood et al., 2022), that has experienced a revival as a commercial crop and is now grown in diverse regions (Hassani et al., 2022; Rodríguez et al., 2024). It belongs to the *Lamiaceae* family, like mint, and about 900 species have been identified within this family under the *Salvia* genus (Ixtaina, 2010; Jash et al., 2016; Ayerza and Coates, 2022). Molecular studies suggest this species belongs to the *Angulatae* clade and is phylogenetically related to S*. leptostachys* Benth and *S. rhyacophila* (Fernald) Eplis (Fragoso-Martínez et al., 2018). The chromosome number of somatic cells of chia is 2n=12, the lowest chromosome number in the genus (Ramamoorthy and Elliott, 1993; Wood et al., 2022). According to its mating system, it is a partially autogamous species, with an outcrossing percentage varying from 1 to 22% depending on the degree of domestication (Hernández-Gómez et al., 2008). Upon maturity, it produces a schizocarp composed of four indehiscent locules which separate to form four partial mericarps known as diaspores, where the seed and fruit constitute a single entity, commonly called seed or nutlet (Ixtaina, 2010; Wood et al., 2022).

Seed preservation is a critical aspect of agriculture and germplasm conservation worldwide, as it ensures the maintenance of biodiversity, food security, and ecosystem resilience. However, seeds are constantly exposed to various unfavorable environmental conditions, including temperature fluctuations, which can significantly impact their viability, longevity, and vigor (TeKrony and Egli, 1991; Zhang et al., 2021). Generally, as the seed storage duration increases, seed vigor gradually declines until complete loss of viability (Bewley and Black, 1994). Temperature stress, both high and low, is among the most influential environmental factors affecting seed preservation (Sano et al., 2016). High temperatures can accelerate the aging process of seeds, leading to reduced germination rates, decreased vigor, and ultimately, loss of seed viability (Reed et al., 2022).

Therefore, understanding the physiological characteristics of chia seeds becomes crucial for preserving their quality, considering their significance in agriculture and nutrition. Researchers have investigated this aspect by evaluating germination capacity, seed viability, and electrical conductivity under various environmental conditions, including temperature, salinity, and seed moisture content. These factors can significantly impact these processes and thus, alter seed quality (Proskura et al., 2018; Radke et al., 2018; Busilacchi et al., 2019; Gonzalez Vera et al., 2019; Sorana et al., 2019; Stefanello et al., 2019; Cabrera-Santos et al., 2021; Cabrera-Santos et al., 2022) Despite providing valuable physiological insights, the seed aging studies conducted so far offer limited information about the cellular mechanisms related to seed deterioration. Consequently, there is a growing interest in identifying molecular markers associated with seed quality, prompting the exploration of new analytical technologies. Among these, untargeted proteomics has emerged as a robust and promising tool for achieving a deeper understanding of the molecular mechanism underlying seed deterioration. Interestingly, this approach is particularly suitable for comparing genotypes that may exhibit differential molecular responses. Genomic (Wang et al., 2022), proteomic (Sandoval-Oliveros and Paredes-López, 2013; Alejo-Jacuinde et al., 2023) and metabolomic (Alejo-Jacuinde et al., 2023; Grauso et al., 2023) studies have been conducted on chia seeds to mainly assess functional food design (Li et al., 2023). However, chia seed deterioration through high-resolution proteomic analysis has not been explored, so it can be considered a novel approach for this type of seed. The 2-DE technique presents limitations in protein separation and identification due to its limited resolution. In contrast, mass spectrometry MS-based proteomics has emerged as a powerful tool for large-scale identification and quantification of proteins (Min et al., 2017; Meyer, 2021; Song et al., 2023). Recent technological advances, such as liquid chromatography coupled with tandem mass spectrometry (LC-MS/MS), have enabled efficient detection and quantification of thousands of proteins in biological samples (Lei et al., 2020; Poza-Viejo et al., 2023; Song et al., 2023).

The main aim of the present work was to analyze the proteome of *Salvia hispanica* L. and evaluate the molecular responses to viability loss induced by high temperature and humidity. To this end, shotgun proteomics followed by liquid chromatography-tandem mass spectrometry (LC-MS/MS) analysis was applied to viable and non-viable chia seeds from two contrasting genotypes in fruit coat color, one mixed (MN) and one white (WN).

## 2 Materials and methods

### 2.1 Plant material and experimental conditions

In this study, two genotypes of chia were examined: one displaying mixed nutlet coloration (white, beige, and greyish brown) (MN) and another, composed exclusively of white nutlets (WN). The MN genotype was provided by the *Estación Experimental Agropecuaria Salta* (INTA), Argentina, and WN was donated by the *Estación Experimental Agroindustrial Obispo Colombres* (EEAOC), Tucumán, Argentina.

The two genotypes were subjected to a controlled storage treatment, hereafter referred to as artificial aging (AA). This treatment involved exposing seeds to specific temperature and humidity conditions to accelerate the aging process. The seeds were stored at 40°C and 100% relative humidity (RH) in a controlled temperature chamber (Ingelab, Buenos Aires, Argentina) for 56 days, which was the time necessary to reach a value of 0% of germination (non-viable seeds). To verify the complete loss of viability after AA, the initial and final viability of the seeds was determined using the tetrazolium test, following the guidelines established by the International Seed Testing Association (ISTA) (International rules for seed testing - international seed testing association).

This design resulted in four treatment groups: seeds from both WN and MN genotypes that were initially viable at the start of the study (T_0_). These seeds represent the initial viable state of WN and MN before AA exposure (WN-T_0_ and MN-T_0_); seeds from both WN and MN genotypes that were subjected to AA and lost viability (WN-AA and MN-AA).

### 2.2 Moisture content

Seed moisture content was measured at 0 and 56 days using the gravimetric method following the low-constant-temperature method proposed by ISTA (International rules for seed testing - international seed testing association). Approximately 1 g of seeds was dried at 103°C for 17 h, and the analysis was performed in duplicate.

### 2.3 Germination test

For germination assessment, four replicates of 25 seeds per lot were first treated with 1% (w/v) sodium hypochlorite and then washed with distilled water. These seeds were placed in Petri dishes containing germination paper (Whatman No: 5, diameter 9 cm). The paper was moistened with distilled water and dishes were sealed with Parafilm®. Subsequently, the Petri dishes were kept in a germination chamber (Ingelab, Buenos Aires, Argentina) with a temperature of 25°C and a photoperiod of 12 h of light and 12 h of darkness. The light intensity was maintained at 45 μmol photons s^-1^m^-2^ using white light. Daily counts were carried out for 5 days, corresponding to the visualization of cotyledons, and a seed was classified as germinated (G) when the radicle exceeded 2 mm in length. At the final count, seedlings were categorized as either normal (NS) or abnormal (AS), according to ISTA (International rules for seed testing - international seed testing association). This analysis was carried out by taking samples every 7 days during the storage period.

The normal germination power (NGP) was calculated as the ratio between NS and G. The mean germination time (MGT) was calculated using Equation 1 (Sadeghi et al., 2011):


(1) $$MGT=\frac{\sum d\;n}{\sum n}$$

where d: germination day counted from the beginning of imbibition; *n*: number of seeds germinated on the day d.

### 2.4 Protein extraction and quantitative label-free proteomic analysis (LC-MS/MS)

#### 2.4.1 Protein extraction

Three biologically independent pools of *S. hispanica* seeds (WN-T_0_; WN-AA; MN-T_0_; MN-AA) were used. The seeds were powdered, and 50 mg of powder from each pool was solubilized in 8 M urea and filtrated to obtain 1 mL of solubilized protein suspension. Protein concentration was measured by fluorometry using the Invitrogen™ Qubit™ 3 (Thermo Fisher Scientific) reagents.

#### 2.4.2 Protein concentration and trypsin digestion

Protein concentration and trypsin digestion were performed as described by Poza-Viejo et al. (2023). In summary, the protein analysis process began with loading 50 µg of each sample onto a 10% acrylamide gel with 4 cm stacking gel using a Mini-PROTEAN® Tetra Cell. Electrophoresis was carried out at 100 V in Laemmli buffer until the electrophoresis front reached 2 cm into the stacking gel. The gels were then fixed in a solution of 50% (v/v) methanol and 2% (v/v) phosphoric acid, followed by washing and rinsing with Milli-Q water. Subsequently, the gels underwent incubation in a mixture of 33% (v/v) methanol, 17% (v/v) ammonium sulfate, and 3% (v/v) phosphoric acid. Protein bands were visualized through overnight incubation in colloidal Coomassie (G-250) and methanol, with subsequent rinsing. Excess Coomassie solution was removed by rinsing the gels with water.

The final steps involved cutting the protein bands. Disulfide bonds were reduced using 20 mM dithiothreitol (DTT) in ammonium bicarbonate 25 mM at 56°C, followed by blocking with 22.5 mM iodoacetamide in ammonium bicarbonate 25 mM in darkness. Two additional ACN washes preceded the gel’s dehydration using SpeedVac for 30 min. Gel slices were rehydrated adding trypsin (1 µg in 20 µl) in ammonium bicarbonate 25 mM for overnight digestion at 37°C. Digested peptides were recovered, dried using SpeedVac for 30 min, and resuspended in a solution of 31 µl ACN 2% (v/v) and formic acid 0.1% (v/v). The peptide concentration was determined using 1 µl of each protein extraction with Invitrogen™ Qubit™ 3.

#### 2.4.3 Reversed-phase liquid chromatography (lc) for peptide separation

One µg of each protein extract was introduced into a nano-HPLC Easy-nLC 1000 (Thermo Fisher Scientific, Massachusetts, United States). Initially, the samples underwent concentration using a precolumn PEPMAP100 C18 NanoViper Trap (Thermo Fisher Scientific, Massachusetts, United States). Subsequently, separation occurred on a 50 cm column PEPMAP RSLC C18 (Thermo Fisher Scientific, Massachusetts, United States) using a gradient of ACN ranging from 5 to 40% (v/v) and formic acid at 0.1% (v/v) over 120 min.

#### 2.4.4 Data-dependent acquisition (DDA) for shotgun proteomics

Peptide fractions were subjected to electrospray ionization in positive mode and examined using a quadrupole Orbitrap mass spectrometer, specifically the Q Exactive HF model (Thermo Fisher Scientific, Massachusetts, United States), operating in DDA mode. Within each mass spectrometry (MS) scan, ranging from 390 to 1700 Da, the 15 most intense precursors with charges between 2+ and 5+ were chosen for high collision energy dissociation (HCD) fragmentation. Subsequently, the corresponding tandem mass spectrometry (MS/MS) spectra were obtained.

### 2.5 Quantitative proteomic analysis

#### 2.5.1 Protein identification

LC–MS/MS data obtained from each chia seed sample were analyzed using Proteome Discoverer 2.4 (Thermo Fisher Scientific, Massachusetts, United States). Peptide-spectrum matches (PSMs) identified each MS/MS spectrum by comparing them to theoretical masses derived from the original precursor mass fragmentation. This comparison utilized the *Salvia hispanica* proteome annotation in the NCBI database (https://www.ncbi.nlm.nih.gov/bioproject/PRJNA830713/). The assigned peptides were linked to annotated *Salvia hispanica* proteins. In cases where a peptide could be assigned to different proteins, the software adhered to the parsimony principle to generate a master protein. The Percolator algorithm was employed to estimate the false discovery rate (FDR). High-confidence proteins were identified and filtered based on a p-adjusted value *(p-adj) < 0.05*.

The mass spectrometry proteomics data have been deposited in the ProteomeXchange Consortium through the PRIDE (Perez-Riverol et al., 2022) partner repository with the dataset identifier PXD049229 and 10.6019/PXD049229.

#### 2.5.2 Peptide and protein normalization

Proteome Discoverer™ 2.4 (Thermo Fisher Scientific, Massachusetts, United States) was employed to assess the abundance of peptides and proteins. Initially, mass recalibration was executed with Sequest HT, comparing the database with identified proteins and aligning chromatography data of the samples with a tolerance of up to 10 min. Subsequently, retention time alignment across all samples was conducted to quantify precursor ions, considering unique peptides present in at least two of the three replicates. Finally, normalization of the total protein amount among samples was achieved using the total abundance of peptides.

#### 2.5.3 Sample pooling and relative protein quantification

For each genotype and aging treatment (comprising non-aged and aged seeds), three biological replicates were examined using a No Nested/Pairwise design. Quantified proteins were derived from peptide ratios, calculated as the geometric median of the peptide ratios within each biological replicate (**Supplementary Tables 1**–**6**).

A two-sample comparison using a background-based *t*-test was performed in Proteome Discoverer 2.4 for the identification of differentially abundant proteins among seed samples, setting a significance level of 0.05. The resulting p-values underwent correction (*p-adj<0.05*) by applying the False Discovery Rate (FDR) through the Benjamin & Hochberg (BH) test (see **Supplementary Tables 1**–**6**). Biological sample distribution was analyzed by principal component analysis (PCA) confirming substantial variation between them (**Supplementary Figure 1**).

#### 2.5.4 Protein functional analysis

The functional analysis of proteins manifesting significant alterations in their relative abundance was carried out using the Kyoto Encyclopedia of Genes and Genomes (KEGG) Mapper - Assign KO web tool (https://www.kegg.jp/kegg/mapper/assign_ko.html). Individual FASTA files were generated, encompassing the sequences of differentially relative abundant proteins in accelerated-aged (AA) seeds compared to non-aged (T0) seeds for each analyzed chia genotype (designated as WN or MN). Subsequently, these FASTA files were submitted to the Assign KO web tool for the assignment of KEGG numbers. The Assign KO tool serves as an interface to the BlastKOALA server, facilitating the allocation of KO identifiers (K numbers) to a given set of sequence data. This assignment is based on mapping the proteins of interest to closely related species. In our investigation, the analysis was restricted to the *Lamiaceae* family dataset with code 4136, to which *Salvia hispanica* belongs (**Supplementary Table 7**).

Fold change values (**Supplementary Table 8**) and volcano plots were represented using the function *ggplot* from R package *ggplot2* v.3.4.4 (R: the R project for statistical computing; Wickham, 2016).

## 3 Results

### 3.1 Effect of artificial aging on chia seed germination and viability

**Figure 1:**
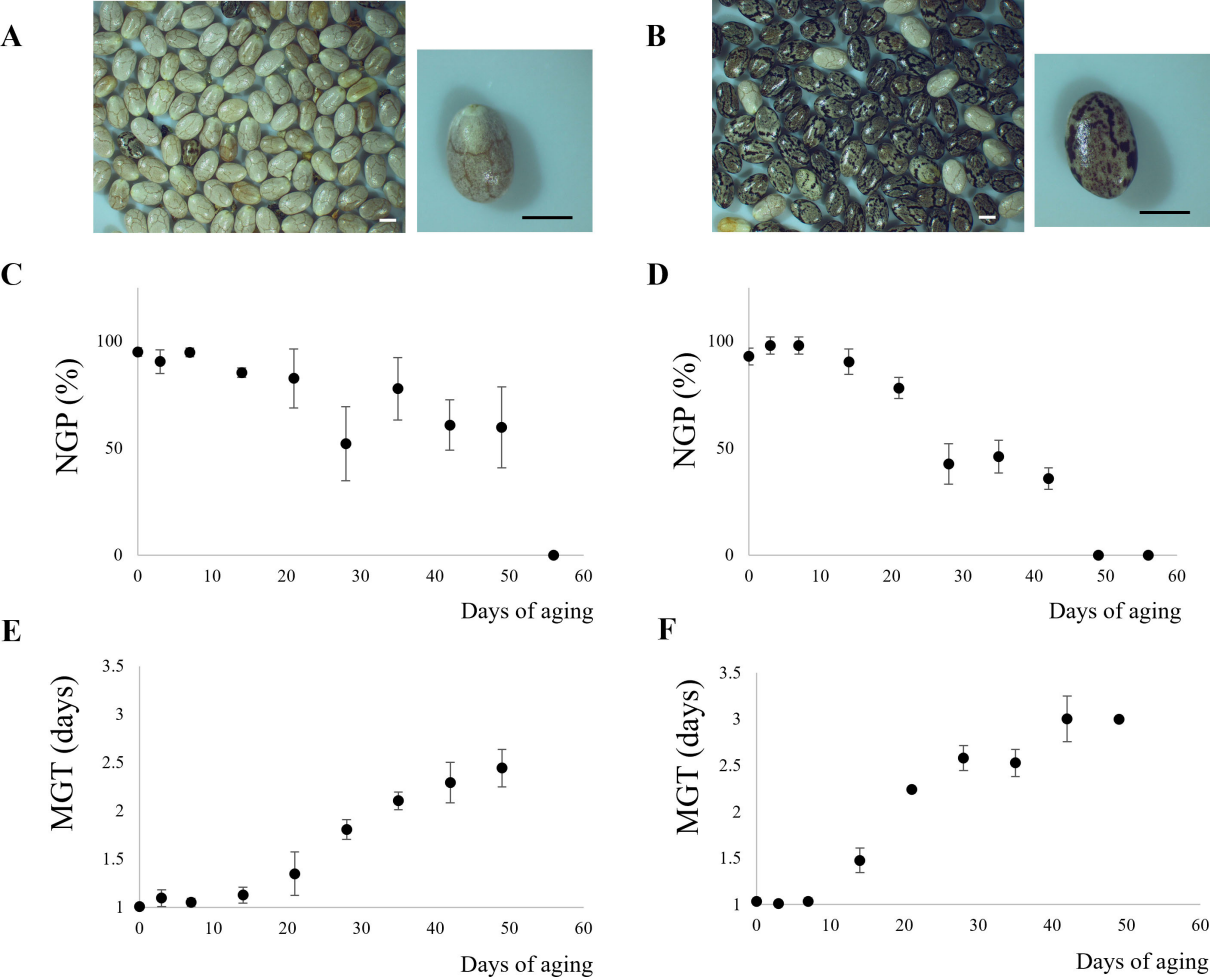
Chia seed physiological response during accelerated aging. Images of chia nutlets of **(A)** WN, and **(B)** MN genotypes. The scale bar represents 1 mm. **(C, D)**, Normal Germination Power (NGP) for **(C)** WN, and **(D)** MN chia genotypes. **(E, F)**, Mean Germination Time (MGT) for **(E)**, WN, and **(F)**, MN nutlets.

The initial moisture content was 4.75% and 3.50% (wet basis, w.b.) for WN and MN, respectively. By the end of the storage period, these values increased to 15.79% (w.b.) for WN and 15.83% (w.b.) for MN. **Figure 1** depicts the impact of aging time on both normal germination power (NGP) and mean germination time (MGT) for WN and MN genotypes. Initially, the NGP for WN and MN was 95% and 91%, respectively, while the initial viability was 100% for WN and 98% for MN. Subsequently, a significant decline (*p< 0.05*) was observed as aging time progressed. In particular, NGP reached zero just after 56 and 49 days of artificial aging for WN and MN genotypes. At these storage times, the viability was 0% for both genotypes. MGT followed a sigmoidal pattern, exhibiting a significant increase (*p< 0.05*) with increasing storage time under high humidity and temperature conditions (**Figure 1**). Initially, MGT was as low as 1 day for both genotypes. However, as NGP approached zero, MGT tended toward infinity. The maximum observed MGT values before complete viability loss were 2.5 and 3 days for WN and MN genotypes, respectively.

### 3.2 Proteomic insights into seed viability loss in chia: genotype-specific differences

**Figure 2:**
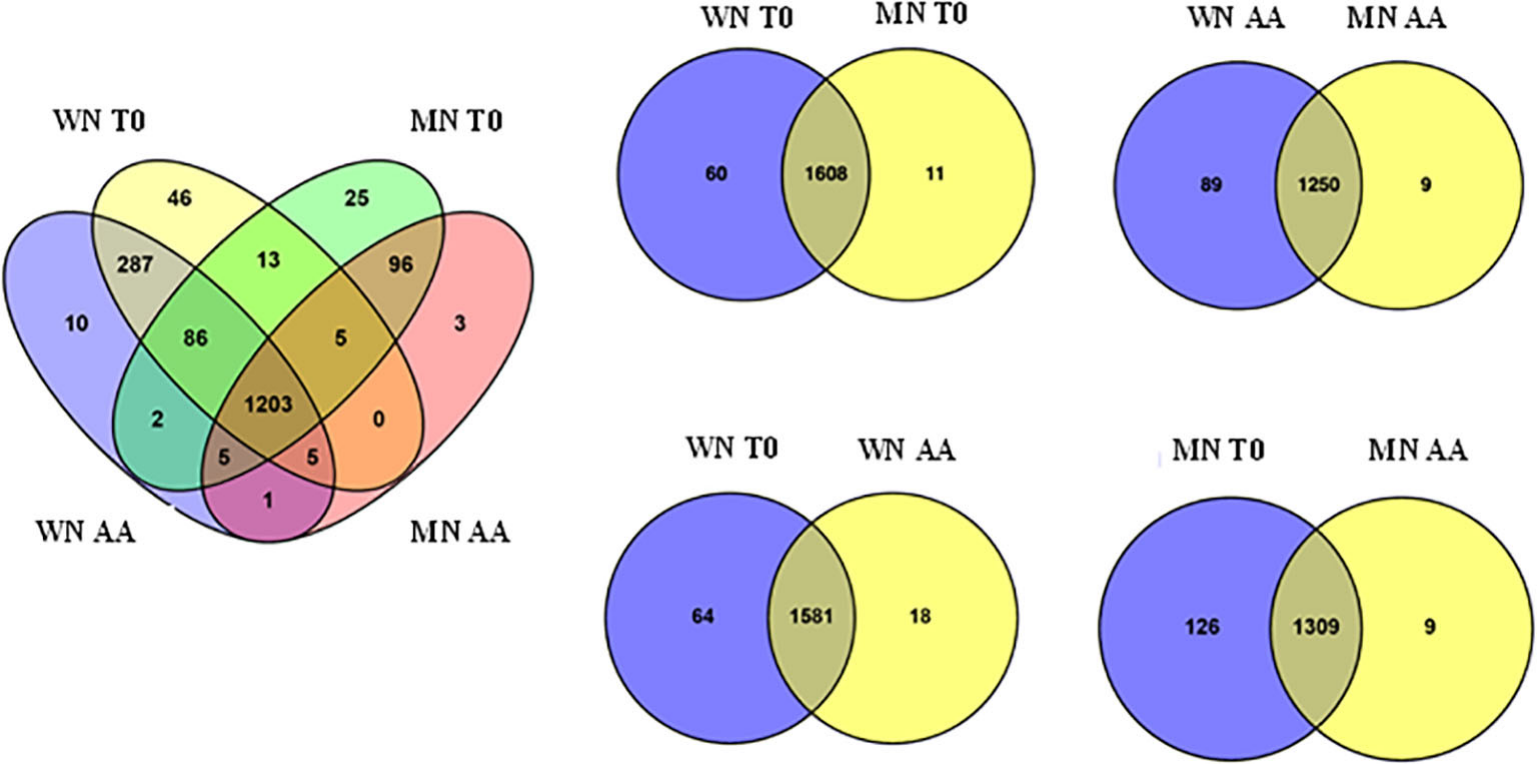
Venn diagrams illustrating the number of identified proteins that are unique and common to the chia genotypes (WN, MN) and aging treatments (AA or T0) analyzed. The left Venn diagram shows all unique and common proteins among genotypes and treatments. Right Venn diagrams show unique and common proteins when comparing by pairs the genotypes (WN or MN) and/or treatments (AA or T0).

To identify protein changes associated with seed viability loss, the global proteome profiles of two contrasting chia coat color genotypes (WN vs MN) were analyzed after 56 days of artificial aging treatment (see Methods section). The proteome analysis identified 1787 proteins at a 95% confidence level. Further analysis of these proteins (**Figure 2**) showed variations based on both genotype and seed viability state (viable vs. non-viable). The analysis revealed genotype-dependent differences in the number of identified proteins, with the WN genotype exhibiting a higher count (1663) than MN (1444). Interestingly, at the initial storage time (T_0_) with viable seeds, 1608 proteins were shared by both genotypes. However, WN displayed a slightly higher number of unique proteins (Delahaie et al., 2013) compared to MN (Rodríguez et al., 2024) (**Figure 2**). In contrast, non-viable seeds resulted in the identification of 1250 common proteins, and 89 and 9 exclusives to WN and MN, respectively. When analyzing the effect of artificial aging in each genotype, the vast majority of proteins were shared between treatments (viable *versus* non-viable) with 1581 and 1309 common proteins found in WN and MN genotypes, respectively. Generally, viable seeds presented a larger number of unique identified proteins than non-viable seeds, with 64 and 126 (for viable WN and MN seeds, respectively) and 18 and 9 (for non-viable WN and MN seeds, respectively).

**Figure 3:**
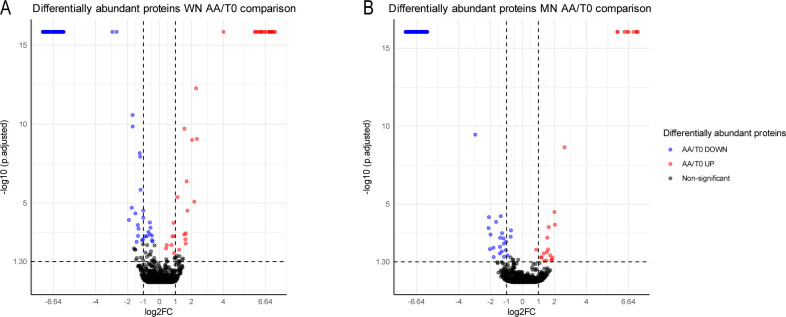
Relative protein abundance of two genotypes of chia seeds (WN and MN) subjected to accelerated aging (AA). **(A)** Volcano plot representing the protein relative abundance when comparing aged versus non-aged seeds for WN genotype. **(B)** Volcano plot representing the protein relative abundance when comparing aged versus non-aged seeds for MN genotype Different colors show two-fold statistically significant overrepresented (red) or down-represented (blue) proteins when comparing AA/CC (| log2FC ≥ 1 |, p-adj ≤ 0.05; n = 3). Black dots represent no statisticallysignificant differentiated proteins

In the current study, protein quantification was performed to determine the differential relative abundance that occurs when going from viable to non-viable conditions after artificial aging (**Figure 3**). In the WN genotype, a significant decrease of 91 proteins and an overaccumulation of 29 were found in non-viable seeds. On the other hand, MN showed 148 proteins decreasing and 26 increasing in non-viable seeds.

**Figure 4:**
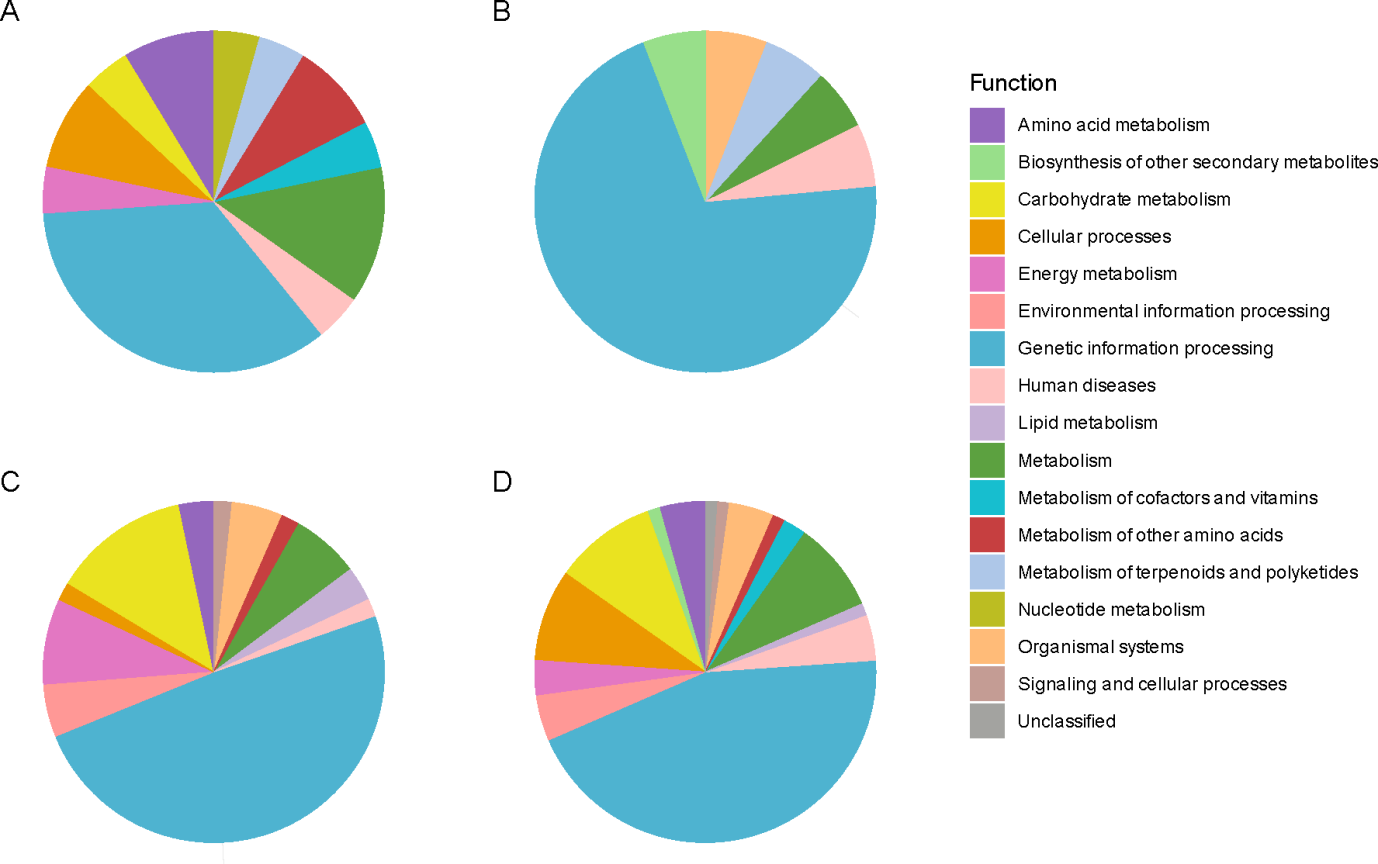
Protein functional analysis of chia seed proteins manifesting significant alterations in their relative abundance. Protein functional analysis was carried out using the Kyoto Encyclopedia of Genes and Genomes (KEGG). This assignment was restricted to mapping the proteins of interest to the Lamiaceae family dataset with code 4136, to which *Salvia hispanica* (chia) belongs. The colors denote distinct KEGG functions to which the identified and quantified proteins were annotated (| log2FC ≥ 1 |, *p-adj ≤ 0.05*; n = 3). Each pie chart illustrates annotated functions for: **(A)** overrepresented proteins in accelerated-aged (AA) compared to non-aged (T0) seeds in the White (WN) genotype, **(B)** overrepresented proteins in AA/T0 in the Mixed (MN) genotype, **(C)** down-represented proteins in AA/T0 in WN genotype, and **(D)** down-represented proteins in AA/T0 in MN genotype.

In the WN genotype, the Kyoto Encyclopedia of Genes and Genomes (KEGG) analysis of Differentially Expressed Proteins (DEP) revealed an enrichment in the abundance of proteins related to the biosynthesis of secondary metabolites (Chaudhary et al., 2021), amino acids (Ebert and Engels, 2020), translation (Lin, 2011), protein folding, sorting, and degradation (Ebert and Engels, 2020), as well as signal transduction (Hrnčič et al., 2020) when the seed lost viability (**Figure 4**; **Supplementary Table 7**). On the contrary, the proteins that showed a decrease in their abundance with the viability loss fell into more diverse functional categories, including the biosynthesis of secondary metabolites (Rodríguez et al., 2024), microbial metabolism in diverse environments (Prathyusha et al., 2019), carbon metabolism (Chaudhary et al., 2021) (including the citrate cycle (TCA cycle) (Lin, 2011) and pyruvate metabolism (Lin, 2011), fatty acid metabolism (Ebert and Engels, 2020), biosynthesis of amino acids (Ebert and Engels, 2020), biosynthesis of nucleotide sugars (Ebert and Engels, 2020), transcription [spliceosome (Hrnčič et al., 2020)], translation (ribosome [Agarwal et al., 2023)] and protein processing in the endoplasmic reticulum (Singh et al., 2022), together with signal transduction (Ayerza and Coates, 2022) and lipid metabolism (Prathyusha et al., 2019), amino acid metabolism (Prathyusha et al., 2019) and oxidative phosphorylation (Lin, 2011), among others.

In the case of the MN genotype, an increase in the abundance of proteins associated with the ribosome (Agarwal et al., 2023) was found as the main enriched functional category. There was also a decrease in proteins related to the biosynthesis of secondary metabolites (Hassani et al., 2022), microbial metabolism in diverse environments (Hrnčič et al., 2020), carbon metabolism (Prathyusha et al., 2019) (including the biosynthesis of amino acids (Singh et al., 2022), the biosynthesis of nucleotide sugars (Ebert and Engels, 2020) and cofactors (Ebert and Engels, 2020) together with glycolysis (Lin, 2011) and citrate cycle (TCA cycle) (Ebert and Engels, 2020) and the amino sugar and nucleotide sugar metabolism (Ebert and Engels, 2020), the glyoxylate and dicarboxylate metabolism (Ebert and Engels, 2020) and the butanoate metabolism (Lin, 2011), the oxidative phosphorylation (Ebert and Engels, 2020) and the amino acid metabolism (Hassani et al., 2022) (**Figure 4**).

**Figure 5:**
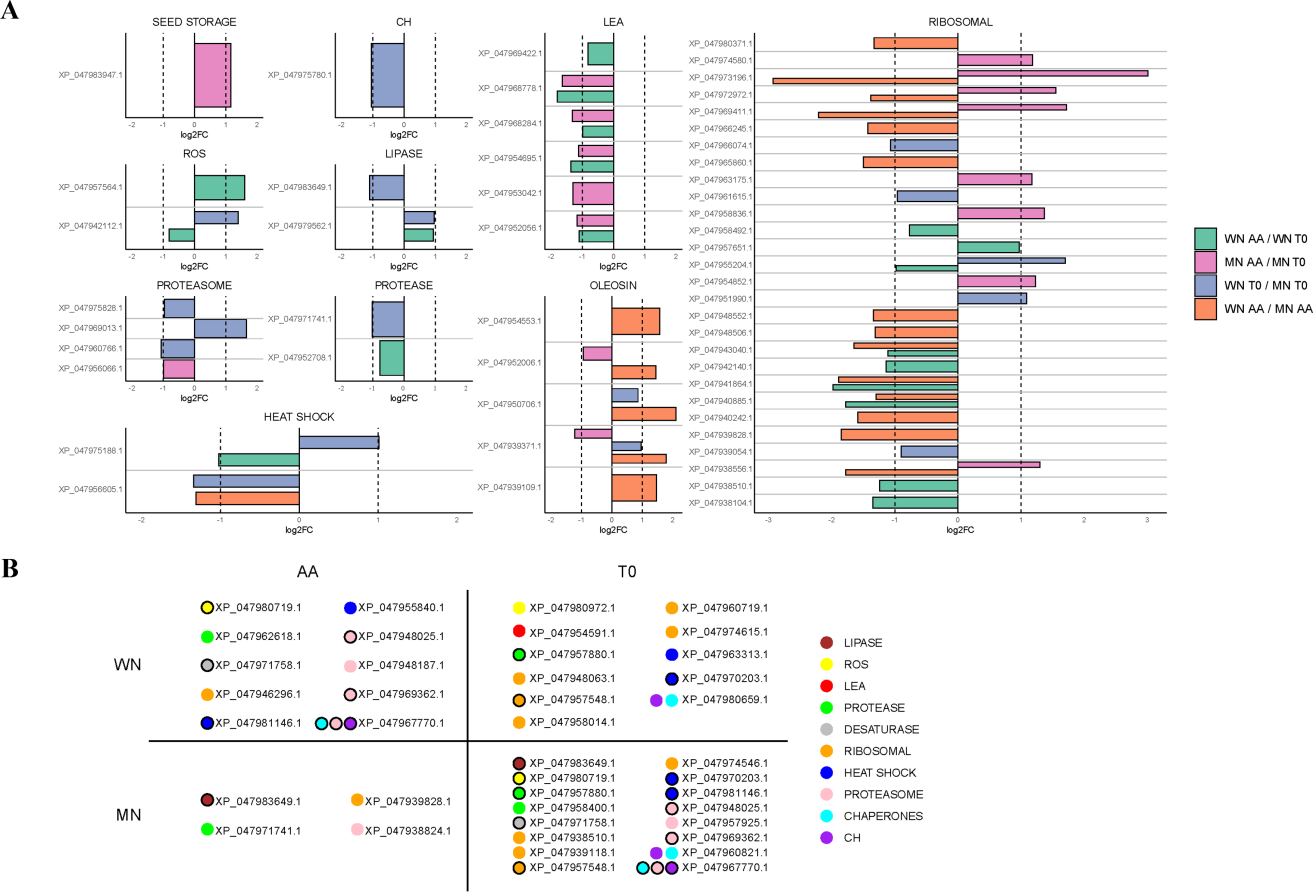
Comparisons of differentially expressed proteins (DEP) among chia genotypes with (AA) or without (T0) aging treatment. **(A)** Various Log2FoldChange (log2FC) comparisons are visually depicted using distinct colors. Differences between genotypes (WN and MN) under the same treatment conditions are indicated in blue for T0 conditions and orange for AA conditions. Additionally, variations between treatment conditions (AA, T0) for each chia genotype are represented in pink for the MN genotype and in green for the WN genotype. The proteins are categorized based on their biological function. **(B)** Exclusive DEP appearing per genotype and treatment. Proteins unique to each chia genotype and treatment. Proteins are color-coded based on their biological function. Specifically, proteins with lipase activity are represented in brown, those related to reactive oxygen species (ROS) in yellow, late embryogenesis abundant proteins (LEA-) in red, protease-related proteins in green, desaturase-related proteins in grey, ribosomal-related proteins in orange, heat shock-related proteins in dark blue, proteasome-related proteins in pink, chaperones in light blue, and carbohydrates-related proteins in purple. Black-bordered circles denote proteins that appear in more than one group, indicating their shared presence between chia genotypes or treatments. Only statistically significant differences, determined using a background-based *t*-test *-*with *p.adj*< 0.05, are shown.

Interestingly, this functional analysis pointed to differential and common elements as responses to viability loss betweengenotypes. For instance, loss of viability in both genotypes resulted in a decreased abundance of a lipoic acid-related protein (KO00785), a cofactor involved in the plant antioxidant response, or proteins related to carbohydrate metabolism (i.e. glycolysis (KO00010) and the TCA cycle (KO00020). However, a distinct response was found at a translational level as MN showed a larger abundance of ribosomal proteins accumulated in its seeds with viability loss. These results were confirmed when classifying and analyzing protein changes according to their nomenclature description (**Figure 5A**; **Supplementary Table 8**). For example, the MN genotype showed a decrease in 2 quantified oleosins (XP_047939371.1 and XP_047952006.1), while in WN no significant changes in this group of proteins were detected (**Figure 5**; **Supplementary Table 8**). Furthermore, the MN genotype was generally accumulating ribosomal proteins compared to WN with 8 proteins being accumulated in this genotype in non-viable seeds (XP_047963175.1, XP_047972972.1, XP_047974580.1, XP_047938556.1, XP_047969411.1, XP_047954852.1, XP_047958836.1, and XP_047973196.1), 1 appearing (XP_047939828.1) and 2 disappearing (XP_047957548.1 and XP_047939118.1). WN showed an opposing response as instead of increasing the abundance, reduced the presence of 8 ribosomal proteins in non-viable seeds (XP_047938510.1, XP_047940885.1, XP_047958492.1, XP_047955204.1, XP_047942140.1, XP_047943040.1, XP_047941864.1, and XP_047938104.1) with also three ribosomal proteins disappearing in non-viable seeds (XP_047957548.1, XP_047958014.1 and XP_047960719.1) (**Figure 5**; **Supplementary Table 8**). Besides, 6 LEA were identified in the WN genotype that decreased in non-viable seeds (XP_047952056.1, XP_047968284.1, XP_047969422.1, XP_047954591.1, XP_047968778.1, XP_047953042.1, and XP_047954695.1), 1 disappearing (XP_047954591.1) and 5 were reduced in MN (XP_047952056.1, XP_047968284.1, XP_047968778.1, XP_047953042.1 and XP_047954695.1) (**Figure 5**; **Supplementary Table 8**).

Regarding enzymes responsible for controlling ROS, one glutathione S-transferase F9-like isoform X2 (XP_047957564.1) was positively regulated for the WN genotype tripling its amount with viability loss. Besides, a superoxide dismutase [Cu-Zn] 2 isoform X1 decreased its content (XP_047942112.1) in this genotype, and another disappeared in non-viable seeds (glutathione S-transferase T1-like, XP_047980972.1). On the other hand, in the MN genotype, a glutathione reductase (XP_047980719.1) was negatively regulated (**Figure 5**; **Supplementary Table 8**).

Lastly, under high-temperature and humidity conditions used in this study, a decrease was observed in all heat shock proteins (HSPs) identified in the WN genotype (XP_047963313.1 and XP_047970203.1 disappearing in non-viable seeds and XP_047975188.1 reducing its amount), supporting their role in maintaining seed viability and vigor (**Figure 5**; **Supplementary Table 8**). Interestingly, in MN seeds 2 HSPs (XP_047970203.1 and XP_047981146.1) also disappeared with terminal aging treatment.

**Figure 6:**
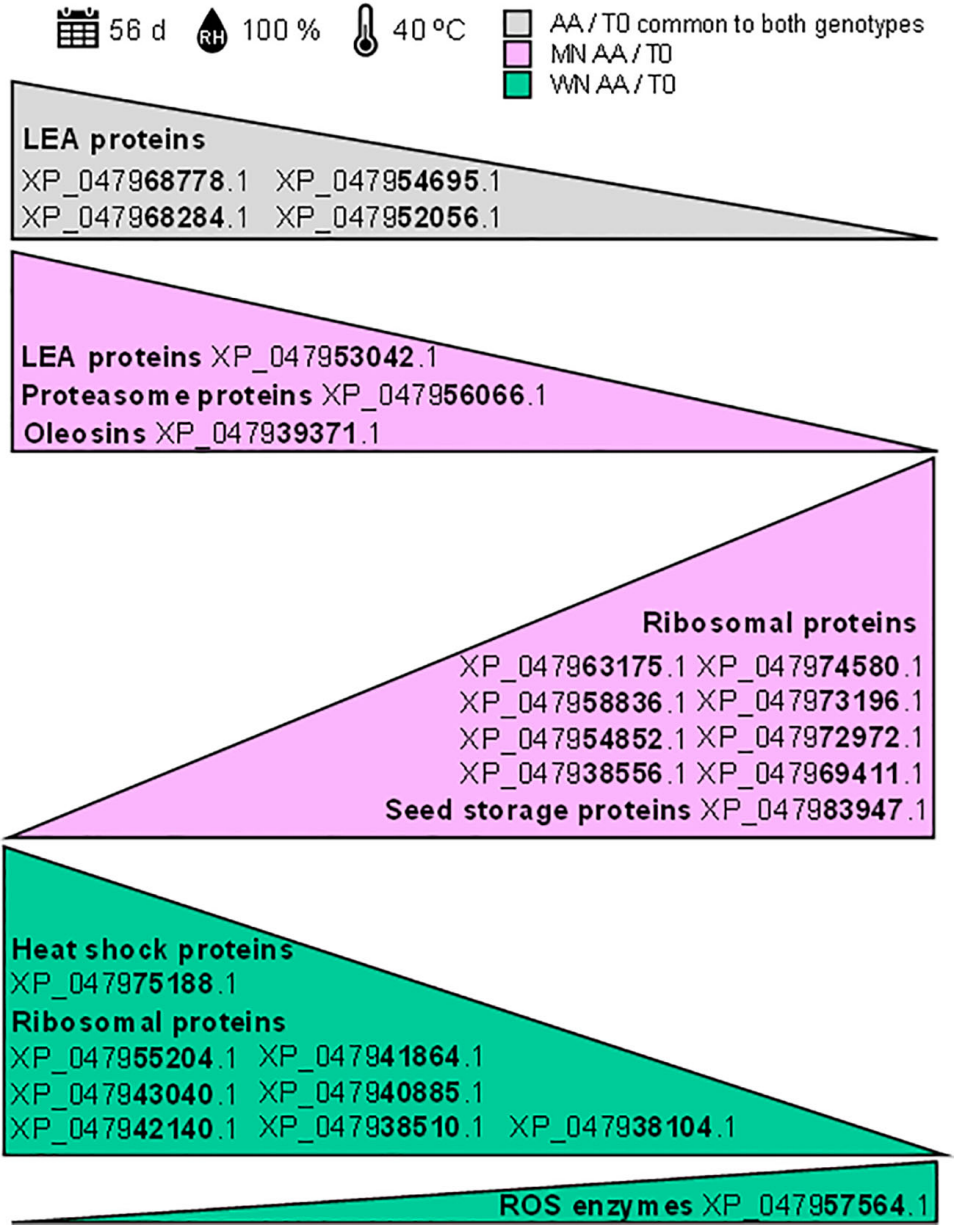
Potential protein biomarkers identified through shotgun proteomics for assessing chia seed viability. The shotgun proteomics analysis revealed potential biomarkers for chia seed viability following exposure to artificial aging (40°C and 100% relative humidity for 56 days). Identified accessions are based on significant Log2FoldChange (log2FC) comparisons using a background-based *t*-test with *p.adj< 0.05*. The grey triangle represents proteins common to both genotypes that decrease under artificial aging conditions (AA). The pink triangles represent proteins that decrease or increase in the MN genotype under artificial aging (AA) conditions. The green triangle represents proteins that decrease in the WN genotype under artificial aging (AA) conditions.

These results reveal that shotgun proteomics unveils potential biomarkers for chia seed viability after exposure to artificial aging, highlighting both shared traits and genotype-specific differences (**Figure 6**). This insight enhances the understanding of seed viability loss response, guiding targeted strategies for seed quality improvement as will be discussed in the next section.

## 4 Discussion

Different biological processes play an essential role in preserving seed longevity. Both endogenous factors (genetic and biochemical) and exogenous factors (humidity and temperature conditions during seed formation, harvest maturity, and subsequent storage) contribute to maintaining the viability and integrity of seeds over time. DNA repair mechanisms, regulation of metabolic activities, and antioxidant production are critical for ensuring seed viability in the long term. Studying and elucidating these molecular mechanisms can improve strategies for preserving chia seeds and contribute to success in conserving genetic resources. The functional annotation KEGG of differentially expressed proteins (DEPs) during aging was analyzed to determine their role in maintaining the viability of chia seeds. Previous studies have evaluated the functionality of proteins in seeds, revealing multiple fundamental DEPs in general metabolism, energy metabolism, protein synthesis, and cellular defense processes during seed aging (Wu et al., 2011; Yin et al., 2015; Xu et al., 2018; Lei et al., 2020). In this study, changes in the accumulation of ribosomal proteins were observed in seeds subjected to conditions of high humidity and temperature, showing contrasting genotypic differences. In yeast, it has been shown that ribosomal RNA (rRNA)-binding proteins form aggregates in response to a decrease in ribosomal DNA (rDNA) stability (Paxman et al., 2022). Additionally, induction of the ribosomal pathway has been detected at the transcriptional and translational levels in aged seeds (86–88). Therefore, the ribosomal pathway could be an indicator of deterioration, possibly involved in regulating seed viability during storage, compromising protein synthesis. The loss of viability of MN genotype seeds could be related to degradative processes mediated by the 26S proteasome, as this genotype showed the disappearance of 5 proteasome-related proteins with aging, reflecting a possible dysfunction in response to environmental stimuli (Xu and Xue, 2019). These hypotheses need to be supported by further research to determine the exact role of ribosomal protein stability in maintaining seed viability, especially since a distinct accumulation pattern was observed between genotypes (MN and WN). Furthermore, viability loss in both genotypes led to a reduction in a protein related to lipoic acid (KO00785) and in proteins involved in carbohydrate metabolism (glycolysis KO00010 and tricarboxylic acid cycle KO00020), compromising vital metabolic functions such as energy provision, essential for all cellular biological processes (Xin et al., 2011).

The longevity in orthodox seeds can be related to the expression of chaperones, such as heat shock proteins (HSPs), proteins abundant during late embryogenesis development (LEA), and the increase in non-reducing sugars and oligosaccharides from the raffinose family (RFO) (Zhou et al., 2020; Ramtekey et al., 2022; Ranganathan and Groot, 2023). These biomolecules confer desiccation tolerance by entering a vitreous state, playing a crucial role in extending longevity due to decreased molecular mobility. Vitrification imposes a highly viscous intracellular environment, limiting residual reactivity and migration of free radicals or reactive oxygen species (ROS) (Buitink et al., 2000; Berjak and Pammenter, 2013). Although the conservation of the intracellular vitreous state is essential for the survival of orthodox seeds, its metastable nature does not confer immortality even under very cold storage conditions, as seed viability decreases in the long term (Berjak and Pammenter, 2013; Walters, 2015).

LEAs, which can be categorized into various groups according to their specific peptides, are small, hydrophilic, thermostable, and unstructured molecules in solution. Although the exact mechanisms through which they protect during desiccation are still speculative, recent proteomic studies have linked specific LEAs with desiccation tolerance in orthodox seeds (Berjak and Pammenter, 2013; Delahaie et al., 2013; Smith and Berjak, 2017). In this study, 6 LEA proteins decreased in the WN genotype during aging, with the disappearance of 1 and the reduction of 5 in MN (**Figure 5**; **Supplementary Table 8**). The high hydrophilicity of LEAs, in combination with sugars, helps stabilize the vitreous state and protect membrane structures during dehydration (Ramtekey et al., 2022). The decrease in these proteins observed in this work was associated with the loss of seed viability, as previously observed in legumes (Ranganathan and Groot, 2023). Therefore, LEAs appear to be involved not only in desiccation tolerance but also in maintaining seed viability during long-term storage, making their monitoring a promising indicator of deterioration. Furthermore, Heat Shock Proteins (HSPs) can promote the folding of polypeptide chains into proteins with natural spatial conformations, as well as the refolding of polypeptides with damaged tertiary structure, thus helping to regulate ROS in seeds (Ul Haq et al., 2019). Thermal stress can induce the formation of HSPs, which would function by repairing proteins denatured by heat (Driedonks et al., 2015; Kaur et al., 2015). In non-viable seeds, all HSPs in the WN genotype decreased (two disappeared, and one was reduced in quantity), indicating an important role in seed viability. Similarly, in MN seeds, two HSPs also disappeared with viability loss (**Figure 5**; **Supplementary Table 8**). These results point to HSPs as proteins sensitive to aging in chia seeds, although further studies are needed to clarify their role in prolonging seed longevity.

Despite the decrease in metabolic activity, the potential for damage caused by reactive oxygen species (ROS) and/or failure of the antioxidant system persists. To counteract this, seeds have enzymatic and non-enzymatic defense mechanisms [investigated in previous studies, i.e (Rodríguez et al., 2024)]. Enzymatic mechanisms include the glutathione metabolism pathway, which maintains the cell’s redox potential, preventing oxidative damage (Song et al., 2023), and the enzymatic pathways of superoxide dismutase (SOD), catalase (CAT), ascorbate peroxidase (APX), and glutathione reductase (GR), which counteract ROS. The WN genotype showed a threefold increase in glutathione S-transferase F9-like in non-viable seeds compared to viable seeds, while superoxide dismutase [Cu-Zn] 2 and glutathione S-transferase T1-like decreased or disappeared, respectively, suggesting an increased oxidative potential. In the MN genotype, GR was downregulated when seeds lost viability (**Figure 5**; **Supplementary Table 8**), suggesting that this genotype could have a more oxidizing redox potential, resulting in damage to subcellular structures and at the level of phospholipid membranes susceptible to ROS attack (Bewley and Black, 1994; Bailly et al., 1997). This result agrees with the physiological assays where NGP and the viability for MN reached a value of 0% a week earlier than WN (**Figure 1**).

Triacylglycerol reserves in seeds (TAGs) are deposited in oil bodies, abundantly present in the embryo of chia seeds (**Supplementary Figure 2**), surrounded by a monolayer of phospholipids in which several unique proteins, especially oleosins, are embedded (D’Andrea, 2016). These proteins appear to be associated with stabilizing this membrane and are crucial during water loss in seed maturation in the mother plant to prevent oil body fusion. Oleosins can also protect phospholipids from hydrolysis by cytoplasmic phospholipases and provide binding sites for lipases involved in TAG mobilization during germination (Board et al., 2022). The chia embryo has a high lipid content that ensures the energy supply for the formation of primary structures of seedlings (**Supplementary Figure 2**). The reduction of oleosins in MN could hinder TAG mobilization, expose phospholipids to phospholipases, and ultimately disrupt oil body structures. All these events could decrease the quality of reserves and, consequently, the energy supply necessary for embryo germination (Shao et al., 2019). Studies on the composition and relative changes in membrane phospholipids could be very helpful in elucidating these mechanisms, as well as the measurement of electrical conductivity to assess the permeability of cell membranes, being a widely used deterioration indicator in germination assays.

Finally, it is worth noting that chia seeds, compared to those of other species (Nigam et al., 2019; Lin et al., 2022), required a longer period for the complete loss of viability under conditions of high temperature and humidity, thus demonstrating remarkable resistance. In this regard, it is necessary to consider the internal structure of the chia nutlet, particularly its pericarp. The pericarp presents a layer of mucilage-secreting cells in the exocarp, followed by a mesocarp with oil-rich parenchyma cells, a layer of macrosclereids, and finally, the endocarp, which contains oil-rich cells covered with wax and underlying layers impregnated with suberin, which forms the inner epidermis of the pericarp (**Supplementary Figure 2**). This structure makes the seeds little permeable to water, evidencing only a 10–12% increase in moisture during aging. Determining vitreous transitions could provide valuable information about the cytoplasm’s aggregation state. Additionally, the thickened layer of the pericarp, positive for safranin and toluidine blue, indicates the presence of lignin, contributing to the “hardness” of the nutlets. Therefore, the observed resistance could be attributed to its diaspore nature, as observed in other plant species. For example, He et al. (2023) found a positive relationship between the presence of pericarp in the fruit of *Nelumbo nucifera* and its remarkable longevity. Although not imperative to prevent water loss in seeds, the pericarp can play a supportive role in conserving seed moisture, contributing to maintaining viability and facilitating germination, as observed in *Swartzia langsdorffii* (Vaz et al., 2018). Thus, among the structural characteristics of chia, the pericarp could be considered a crucial factor in maintaining seed viability during storage.

Considering the results here presented, it has been demonstrated that key proteins, such as late embryogenesis abundant (LEA) proteins, experienced significant changes during the artificial aging process (**Figure 6**). The notable decrease in LEA proteins (accession numbers: XP_047968778.1, XP_047968284.1, XP_047954695.1, XP_047952056.1, and XP_047953042.1) suggests their essential role in maintaining seed viability under stress conditions. Additionally, alterations in the patterns of ribosomal proteins were observed, with variable levels of reduction indicating genotype-specific responses to environmental stress, potentially related to differences in the degree of deterioration. Among these are ribosomal proteins with accession numbers XP_047963175.1, XP_047958836.1, XP_047954852.1, XP_047938556.1, XP_047974580.1, XP_047973196.1, XP_047972972.1, XP_047969411.1, XP_047955204.1, XP_047943040.1, XP_047942140.1, XP_047941864.1, XP_047940885.1, XP_047938510.1, and XP_047938104.1. Moreover, significant modifications were observed in storage proteins, oleosins, and enzymes related to reactive oxygen species (ROS) (accession numbers: XP_047983947.1, XP_047939371.1, and XP_047957564.1), some of which were genotype-specific, underscoring their role in protecting cellular integrity. These findings provide valuable insights into the proteomic alterations associated with seed viability and open new perspectives for improving storage strategies under adverse conditions. Regardless of whether the use of targeted proteomics is necessary to validate these changes, the identified biomarkers hold significant potential for assessing and enhancing seed viability.

## 5 Conclusions

The application of targeted proteomics in this study has identified potential biomarkers of chia seed viability after exposure to high temperature and humidity conditions, revealing differences and similarities between the two genotypes analyzed. This exhaustive analysis, utilizing label-free quantitative LC-MS/MS, allowed the identification of 1787 proteins with a confidence level of 95%, resulting in the most comprehensive chia seed proteome published to date. Among the identified proteins were storage proteins, HSPs, LEAs, oleosins, antioxidant enzymes, and ribosomal proteins.

Although most proteins were shared between the different conditions, significant differences in the abundance of specific proteins were observed between genotypes. The WN genotype presented 130 differentially abundant proteins, while MN showed changes in 174 proteins. Gene Ontology (GO) terms and functional analyses revealed variations in essential protein functions and a significant decrease in proteins responsible for maintaining seed functionality, longevity, and vigor, including storage proteins, LEAs, HSPs, and antioxidant enzymes. Additionally, divergent responses were observed in ribosomal proteins, which accumulated in MN and decreased in WN, as well as negative regulation of proteasome-related proteins exclusively in MN.

Overall, these findings represent a crucial starting point for future research on proteomic changes in the early stages of artificial aging and naturally aged genotypes. This evidence will pave the way for a deeper understanding of identifying deterioration markers and the stress response in stored seeds, which will have a significant impact on seed storage and genetic conservation.

## Data Availability

The datasets presented in this study can be found in the following online repositories: ProteomeXchange with identifier PXD049229.

## Supplementary material

Supplementary Table 1 Protein identification and quantification comparing aged (AA) and non-aged (T0) chia seeds.

Supplementary Table 2 Protein identification and quantification comparing chia genotypes (WN and MN) of aged (AA) seeds.

Supplementary Table 3 Protein identification and quantification comparing seed proteins of MN genotype aged (AA) and non-aged (T0).

Supplementary Table 4 Protein identification and quantification comparing chia genotypes (WN and MN) of non-aged (T0) seeds.

Supplementary Table 5 Protein identification and quantification comparing chia seeds by the genotype (WN and MN).

Supplementary Table 6 Protein identification and quantification comparing seed proteins of WN genotype aged (AA) and non-aged (T0).

Supplementary Table 7 Functional annotation using the Kyoto Encyclopedia of Genes and Genomes (KEGG) of Differentially Expressed Proteins (DEP) during accelerated aging for each chia genotype (WN or MN).

Supplementary Table 8 List of fold change values of proteins identified as differentially abundant between samples (**Figure 5**).
