# Supplementary material for: Shotgun proteomics profiling of chia seeds (Salvia hispanica L.) reveals genotypic differential responses to viability loss
Source: Front Plant Sci. 2024 Aug 15;15:1441234. doi: 10.3389/fpls.2024.1441234 (PMC11358080; doi:10.3389/fpls.2024.1441234)
Supplement: Supplementary file 1 [file Presentation1.pptx]

## Slide 1
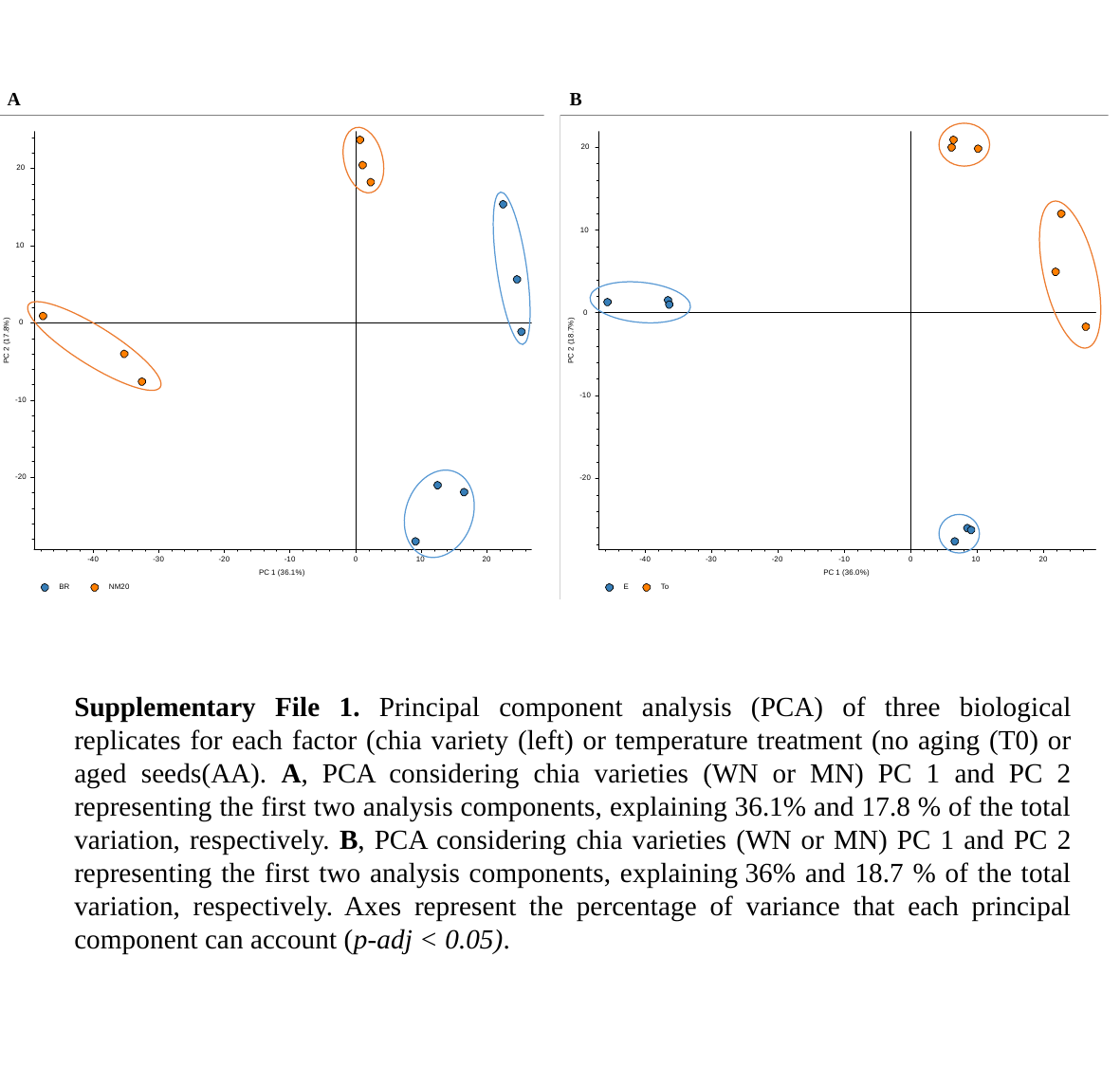

Supplementary File 1. Principal component analysis (PCA) of three biological replicates for each factor (chia variety (left) or temperature treatment (no aging (T0) or aged seeds(AA). A, PCA considering chia varieties (WN or MN) PC 1 and PC 2 representing the first two analysis components, explaining 36.1% and 17.8 % of the total variation, respectively. B, PCA considering chia varieties (WN or MN) PC 1 and PC 2 representing the first two analysis components, explaining 36% and 18.7 % of the total variation, respectively. Axes represent the percentage of variance that each principal component can account (p-adj < 0.05).
