# Supplementary material for: Shotgun proteomics profiling of chia seeds (Salvia hispanica L.) reveals genotypic differential responses to viability loss
Source: Front Plant Sci. 2024 Aug 15;15:1441234. doi: 10.3389/fpls.2024.1441234 (PMC11358080; doi:10.3389/fpls.2024.1441234)
Supplement: Supplementary file 2 [file Presentation2.pptx]

## Slide 1
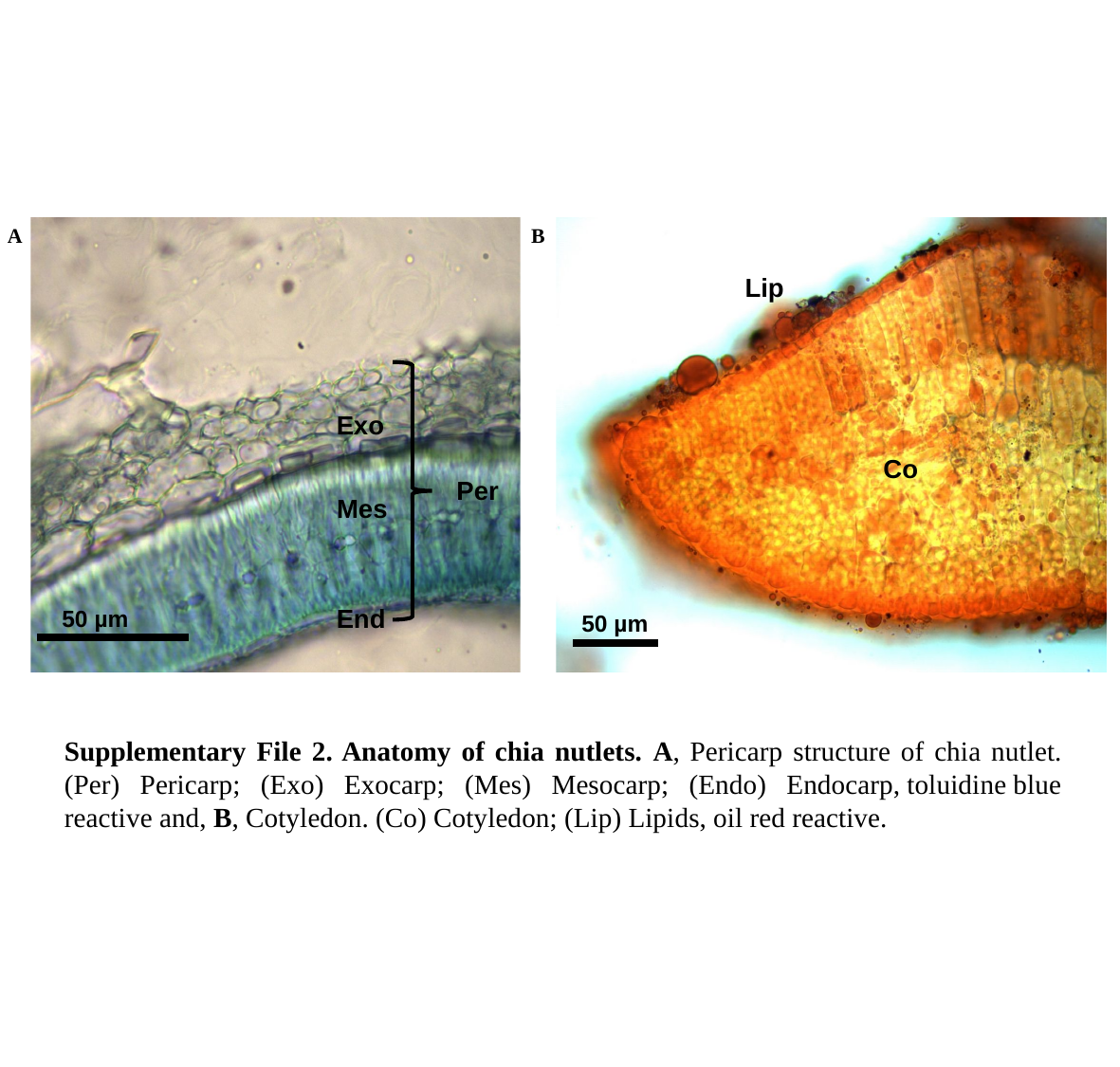

Supplementary File 2. Anatomy of chia nutlets. A, Pericarp structure of chia nutlet. (Per) Pericarp; (Exo) Exocarp; (Mes) Mesocarp; (Endo) Endocarp, toluidine blue reactive and, B, Cotyledon. (Co) Cotyledon; (Lip) Lipids, oil red reactive.
